# Flavonoid Hesperidin Induces Synapse Formation and Improves Memory Performance through the Astrocytic TGF-β1

**DOI:** 10.3389/fnagi.2017.00184

**Published:** 2017-06-13

**Authors:** Isadora Matias, Luan P. Diniz, Andrea Buosi, Gilda Neves, Joice Stipursky, Flávia Carvalho Alcantara Gomes

**Affiliations:** Instituto de Ciências Biomédicas, Universidade Federal do Rio de JaneiroRio de Janeiro, Brazil

**Keywords:** astrocyte, synapse, hesperidin, flavonoids, memory, TGF-β1

## Abstract

Synapse formation and function are critical events for the brain function and cognition. Astrocytes are active participants in the control of synapses during development and adulthood, but the mechanisms underlying astrocyte synaptogenic potential only began to be better understood recently. Currently, new drugs and molecules, including the flavonoids, have been studied as therapeutic alternatives for modulation of cognitive processes in physiological and pathological conditions. However, the cellular targets and mechanisms of actions of flavonoids remain poorly elucidated. In the present study, we investigated the effects of hesperidin on memory and its cellular and molecular targets *in vivo* and *in vitro*, by using a short-term protocol of treatment. The novel object recognition test (NOR) was used to evaluate memory performance of mice intraperitoneally treated with hesperidin 30 min before the training and again before the test phase. The direct effects of hesperidin on synapses and astrocytes were also investigated using *in vitro* approaches. Here, we described hesperidin as a new drug able to improve memory in healthy adult mice by two main mechanisms: directly, by inducing synapse formation and function between hippocampal and cortical neurons; and indirectly, by enhancing the synaptogenic ability of cortical astrocytes mainly due to increased secretion of transforming growth factor beta-1 (TGF-β1) by these cells. Our data reinforces the known neuroprotective effect of hesperidin and, by the first time, characterizes its synaptogenic action on the central nervous system (CNS), pointing astrocytes and TGF-β1 signaling as new cellular and molecular targets of hesperidin. Our work provides not only new data regarding flavonoid’s actions on the CNS but also shed light on possible new therapeutic alternative based on astrocyte biology.

## Introduction

Synapse formation and plasticity are key properties throughout the life of animals and are crucial to their cognitive abilities, such as learning and memory. In contrast, synaptic dysfunctions are involved in the pathogenesis of several neurological conditions, particularly neurodegenerative and psychiatric disorders (Buffington et al., 2014). Currently, strong evidences point to glial cells, especially astrocytes, as active participants in synapse formation and function during development and adulthood (Eroglu and Barres, 2010; Diniz et al., 2014a). Conversely, astrocyte dysfunctions are also present in brain diseases and may drive synaptic alterations and cognitive impairments in these cases (Chung et al., 2015). Nevertheless, cellular and molecular mechanisms underlying the astrocytic control of synapses in health and disease are only now beginning to be better understood.

Astrocytes closely and dynamically control synapses by two known mechanisms: expression of adhesion molecules (Hama et al., 2004) and secretion of soluble factors, such as cholesterol (Mauch et al., 2001), thrombospondin 1 (Christopherson et al., 2005), hevin (Kucukdereli et al., 2011), glypicans (Allen et al., 2012) and transforming growth factor beta-1 (TGF-β1; Diniz et al., 2012). Recently, elegant studies have pointed to the critical role of some of these molecules secreted by adult murine and human astrocytes in the formation of functional synapses (Han et al., 2013; Zhang et al., 2016). Genetic inhibition of vesicular release from astrocytes, a pathway also involved in the secretion of synaptogenic soluble factors, strongly affects the synaptic integration of adult-born hippocampal neurons (Sultan et al., 2015). Although the secretion profile of astrocytes has been investigated recently (Orre et al., 2014; Zhang et al., 2016), the mechanisms involved in the control of astrocyte secretion are still poorly known.

Natural compounds have been successfully discovered and used in “popular medicine” for thousands of years, and their mechanisms of action and therapeutics have been better described from the past decades (Ji et al., 2009). Flavonoids constitute the richest class of polyphenolic compounds in nature, with more than 4000 varieties identified. They are widely found in fruits, leaves, grains, bark, roots, stems, flowers and even in plant-derivatives such as tea and wine. They are classified into four main groups according to their molecular structures: flavones, flavanones, catechins and antocyanins (Nijveldt et al., 2001).

Currently, a growing number of clinical trials have revealed beneficial effects of flavonoids on the human brain. Adoption of a high cocoa flavanol-containing diet can ameliorate cognitive function in elderly humans (Brickman et al., 2014), as well as a higher intake of anthocyanidins, mainly found in berries, reduces cognitive decline in older women (Devore et al., 2012). It has also been shown that chronic consumption of flavanone-rich orange juice by healthy older adults improved their cognitive function (Kean et al., 2015).

Most of the beneficial effects of flavonoids on cognition in animal models have been related to their antioxidant activity and their ability to control neuronal function, survival, synaptic plasticity and long-term potentiation (LTP; Bhullar and Rupasinghe, 2013). Indeed, neuronal-binding sites and signaling pathways have been described to be modulated by flavonoids. It is well know that isoflavones, such as genistein and daidzein, behave as phytoestrogens and are able to interact and activate estrogen receptors (Rickard et al., 2003; Adams et al., 2012). Besides, the first flavonoid described as an agonist of TrkB receptors was 7,8 dihydroxyflavone, which is able to modulate neuronal survival and function through the activation of this pathway (Jang et al., 2010; Zhang et al., 2014). Despite this information, flavonoid’s cellular targets and molecular mechanisms of action remain poorly understood, especially concerning their actions on non-neuronal cells. Glial cells are highly responsive to flavonoids (Matias et al., 2016), as these compounds may: (1) modulate the redox state of astrocytes through an up-regulation of antioxidant enzymes and genes (Bahia et al., 2008); (2) reduce neuroinflammation by controlling astrocyte reactivity and secretion of pro-inflammatory cytokines (Khan et al., 2016; Rehman et al., 2017); and (3) regulate the secretion of trophic factors by astrocytes (Xu et al., 2013a; Wang et al., 2014).

We previously showed that hesperidin, the major flavanone glycoside present in citrus fruits (Garg et al., 2001), promotes neuronal differentiation and survival (Nones et al., 2011) and also enhances the neuroprotective capacity of astrocytes, by inducing them to secrete soluble factors involved in neuronal survival *in vitro* (Nones et al., 2012b). Nevertheless, the identity of astrocyte-secreted factors induced by hesperidin remains unknown, as well as its impact on astrocyte function.

Here, we hypothesized that hesperidin modulates cognitive ability of healthy adult mice by affecting the synaptogenic potential of astrocytes. By using different experimental approaches, we showed that the short-term treatment with the flavonoid ameliorates the memory performance of mice, which was followed by an increase in the density of hippocampal synapses *in vivo*. *In vitro* data indicated that this was mainly due to: (1) direct promotion of synapse formation and activity; and (2) induction of TGF-β1 secretion and its signaling pathway activation in astrocytes. Therefore, our work reveals new data regarding the actions of flavonoids in the central nervous system (CNS) and their cellular and molecular mechanisms underlying synapse formation.

## Materials and Methods

### Animals

Embryonic day 14–15 and newborn (P0) Swiss mice were used for neuronal and astrocyte cultures, respectively. For *in vivo* experiments, we used 3-month-old male Swiss mice (CECAL, Fiocruz breeding colony). Adult animals were housed in groups of 5 mice in plastic cages (17 × 28 × 13 cm) with free access to certified food (Nuvital^®^) and tap water. Mice were kept at controlled room temperature (24 ± 2°C) and humidity, under a 12 h light-dark cycle (lights off at 6 pm) and were adapted to local conditions for at least 1 week before the experiments. All procedures were previously approved by the local Animal Care Ethical Committee (CEUA-UFRJ, approval protocols DFBCICB053 and 004/16) and performed according to Brazilian Guidelines on Care and Use of Animals for Scientific and Teaching Purposes (DBCA), National Council for Animal Experimentation Control—CONCEA, 2013, and to Directive of the European Parliament and of the Council of the European Union of 22 September 2010 (2010/63/EU).

### Drugs

The flavonoid hesperidin (C28H34O15, CAS number 520-26-3) was purchased from Sigma-Aldrich (St. Louis, MO, USA). For cell culture assays, hesperidin was diluted in dimethyl sulfoxide (DMSO; Sigma Chemical Co., St. Louis, MO, USA) and used at a final concentration of 5 μM or 10 μM, as specified bellow. For *in vivo* experiments, hesperidin was prepared as previously described (Donato et al., 2014). Hesperidin was diluted in DMSO at a final concentration of 5%, a solution of 0, 25% polysorbate 80 at a final concentration of 20% and in saline solution to complete the total volume.

### Experimental Design

#### Drug Administration and Novel Object Recognition Test

The novel object recognition test (NOR) is one of the most widely used behavioral tests to evaluate recognition memory in mice. We used a modified protocol from Antunes and Biala (2012), as described below: after 3 days of habituation sessions (10 min/day, low light condition), mice were treated intraperitoneally (i.p.) with hesperidin (10 mg/kg, *n* = 9 animals) or vehicle (control group, 10 mL/kg, *n* = 9 animals), 30 min before the training session. During this session, mice were placed in a circular arena (40 cm diameter, 30 cm high) in the presence of two equal objects for 10 min. After 48 h, they received one more i.p. injection of hesperidin or vehicle 30 min before the 5-min-long test session. Then, animals were placed back in the arena in which one of the objects was replaced by a novel one, unfamiliar object. The arena and objects were cleaned thoroughly between trials with 10% ethanol to eliminate olfactory cues. The time spent by the animals exploring the objects was recorded. Exploratory behavior was defined as sniffing or touching the objects with the front paws or nose. Total traveled distance (cm) and the mean locomotor velocity (cm/s) animals were evaluated in both sessions using MouseGlob software.

#### Immunohistochemistry and Quantification of Synaptic Markers

The animals were anesthetized i.p. with ketamine (100 mg/kg) and xylazine (10 mg/kg) and transcardially perfused with saline solution. Brains were removed and one hemisphere of each brain was fixed with 4% paraformaldeyde (PFA) for 24 h. Serial 10 μm saggital cryosections were mounted on glass slides and submitted to immunofluorescence labeling. Briefly, cryosections were dried, washed in Tris-buffered saline (TBS), permeabilized in 0.5% Triton X-100 for 30 min and then blocked in TBS containing 0.2% Triton-X, 5% goat serum (Invitrogen, Carlsbad, CA, USA) and 3% of BSA (Sigma Chemical Co., St. Louis, MO, USA) for 1 h at room temperature before incubation with the synaptic primary antibodies: mouse anti-synaptophysin (Chemicon International, Billerica, MA, USA; 1:1000), and rabbit anti-Drebrin A/E (Millipore, Darmstadt, DE; 1:1000) overnight at 4°C. Secondary antibodies were Alexa Fluor 546-conjugated goat anti-rabbit IgG or goat anti-mouse IgG (Molecular Probes, Paisley, UK; 1:1000), or Alexa Fluor 488-conjugated goat anti-rabbit IgG or goat anti-mouse IgG (Molecular Probes, Paisley, UK; 1:300). Nuclei were counterstained with 4′,6-diamidino-2-phenylindole dihydrochloride (DAPI; Sigma Chemical Co., St. Louis, MO, USA) and coverslips were mounted with Dako Mounting Media.

Hippocampal CA1 stratum radiatum regions were analyzed on a confocal microscope (Leica TCS SPE) with a 63× oil objective, using the same image parameters for all experimental groups. Synaptic density was evaluated by the Puncta Analyzer plug-in in NIH Image-J as previously described (Diniz et al., 2014b). For each confocal experiment (the comparison of synaptic markers between control and hesperidin groups), we imaged 2–4 equidistant brain tissue sections per mouse, with 2–4 images per section. Results represent the mean of four and three independent animals for control and hesperidin group, respectively.

#### Immunoblotting Assays

Protein concentration, in cell and hippocampal tissue extracts, was measured by the BCA™ Protein Assay Kit (Cole-Parmer Canada Inc., Montreal, QC, Canada). Forty micrograms of protein per lane were submitted to electrophoresis in a 10% SDS-PAGE gel and electrically transferred onto a Hybond-P PVDF transfer membrane (Millipore, Darmstadt, DE) for 1.5 h. Membranes were blocked in phosphate-buffered saline (PBS)-milk 5% for 1 h at room temperature. Next, membranes were incubated in block solution overnight in the presence of the following antibodies: mouse anti-synaptophysin (Chemicon International, Billerica, MA, USA; 1:1000), rabbit anti-postsynaptic density protein 95 (anti-PSD-95; Abcam, Cambridge, MA, USA; 1:1000), rabbit anti-Drebrin A/E (Millipore, Darmstadt, DE; 1:1000), rabbit anti-α-amino-3-hydroxy-5-methyl-4-isoxazolepropionic acid receptor (anti-AMPAR; Abcam, Cambridge, MA, USA; 1:1000), rabbit anti-glial fibrillary acidic protein (anti-GFAP; Dako, Cytomation, Glostrup, Denmark; 1:5000), rabbit anti-phospho-SMADs 2/3 (Santa Cruz Biotechnology, Santa Cruz, CA, USA; 1:200), mouse anti-glyceraldehyde-3-phosphate dehydrogenase (anti-GAPDH; Abcam, Cambridge, MA, USA; 1:1000), mouse anti-α-Tubulin (Sigma Chemical Co., St. Louis, MO, USA; 1:5000), rabbit anti-β-actin (Abcam, Cambridge, MA, USA; 1:1000). Membranes were incubated for 1 h with IRDye 680CW goat anti-mouse antibody and IRDye 800CW goat anti-rabbit antibody (LI-COR, Lincoln, USA; 1:20,000). After washing, membranes were scanned and analyzed using Un-Scan-It gel version 6.1 (Silk Scientific, Inc., Orem, UT, USA).

#### Neuronal Cultures and Treatment

Neuronal cortical cultures were prepared as described previously by our group (Diniz et al., 2012). Briefly, the cerebral cortices of embryonic day 14–15 Swiss mice were removed, meninges were carefully removed, neural tissue was dissociated in Neurobasal medium (Invitrogen, Carlsbad, CA, USA) and the cells were plated at a density of 75,000 per well of 13 mm diameter and 500,000 cells per well of 35 mm diameter, onto glass coverslips previously coated with poly-L-lysine (10 μg/mL, Sigma Chemical Co., St. Louis, MO, USA). Cultures were maintained in Neurobasal medium supplemented with B-27, penicillin, streptomycin, fungizone, L-glutamine and cytosine arabinoside (0.65 μM, Sigma Chemical Co., St. Louis, MO, USA). Cultures were kept at 37°C in a humidified 5% CO_2_, 95% air atmosphere for 6 days or 12–14 days *in vitro* (DIV).

Neuronal cultures were submitted to three different type of treatments and in all of them hesperidin was used at a final concentration of 5 μM: (1) to analyze cell death, cultures were treated on the 3 DIV for 72 h; (2) to analyze synapse formation, cultures were treated for 24 h on the 12 DIV; and (3) to analyze synaptic activity, cultures were incubated with hesperidin throughout the 12 DIV.

#### Murine Astrocyte Cultures and Treatment

Primary cortical astrocyte cultures were derived from newborn Swiss mice as previously described (Diniz et al., 2012). Briefly, the mice were decapitated, the cerebral cortices were removed and the meninges were carefully stripped off. Tissues were maintained in Dulbecco’s minimum essential medium (DMEM) and nutrient mixture F12 (DMEM/F12, Invitrogen, Carlsbad, CA, USA), enriched with glucose (3.3 × 10^−2^ M), glutamine (2 × 10^−3^ M) and sodium bicarbonate (0.3 × 10^−2^ M) and dissociated into single cells. Dissociated cells were plated onto glass coverslips in a 24-well plate (Corning Incorporated, Corning, NY, USA), previously coated with poly-L-lysine (Sigma Chemical Co., St. Louis, MO, USA), in DMEM/F12 medium supplemented with 10% fetal bovine serum (FBS; Invitrogen, Carlsbad, CA, USA). The cultures were incubated at 37°C in a humidified 5% CO_2_, 95% air atmosphere. Cell culture medium was changed 24 h after plating and, subsequently, every 2 days, until reaching confluence, which usually occurred after 7–10 days. After that, cells were subjected to one passage to generate purified astrocyte cultures (secondary cultures), which was constituted by more than 95% of GFAP-positive cells.

Astrocytes were treated with 10 μM hesperidin, as previously described (Nones et al., 2011, 2012b), for different periods (0, 30 min and 2 h) for kinetic analysis of nuclear translocation and phosphorylation of SMADs 2/3.

#### Astrocyte-Conditioned Medium (ACM)

Confluent secondary astrocyte cultures were washed to eliminate residual serum and incubated for an additional day in DMEM/F12 serum-free medium. Then, the culture medium was replaced by DMEM/F12 supplemented with 10 μM hesperidin to generate astrocyte-conditioned medium (ACM)-Hesperidin, or 0.1% DMSO, ACM-Control, and kept for 24 h. After that, the cultures were washed and the medium was replaced by serum-free medium and maintained for additional 24 h. Those ACM were collected, centrifuged at 1000× *g* ×10 min to remove cellular debris and used immediately or stored in aliquots at −70°C for further use.

To investigate the effect of ACM on synapse formation, 12 DIV-neuronal cultures were treated with the ACM-Control or ACM-Hesperidin for 3 h. For the neutralization of TGF-β1 activity in the ACMs, the medium was pre-incubated with 1 μg/mL of neutralizing antibody against TGF-β1 (Abcam, Cambridge, MA, USA) for 30 min at room temperature. After that, neuronal cultures were simultaneously maintained in the presence of ACM and the neutralizing antibody for 3 h, followed by fixation and immunostaining for synaptic proteins.

#### Immunocytochemistry

Cultured cells were fixed with 4% PFA for 10 min and permeabilized with 0.2% Triton X-100 for 5 min at room temperature. After, the cells were blocked with 3% bovine serum albumin and 5% normal goat serum (Sigma, St. Louis, MO, USA) in PBS (blocking solution) for 1 h and, then, incubated overnight at 4°C with the specified primary antibodies diluted in blocking solution. The primary antibodies were mouse anti-β-tubulin III (Promega, Madison, WI, USA; 1:1000), rabbit anti-cleaved caspase-3 (Cell Signaling, Beverly, MA, USA; 1:50), rabbit anti-GFAP (Dako Corporation, Glostrup, Denmark; 1:1000), mouse anti-synaptophysin (Chemicon International, Billerica, MA, USA; 1:1000), rabbit anti-PSD-95 (Cell Signaling Technology, Beverly, MA, USA; 1:100), mouse anti-SMADs 2/3 (Santa Cruz Biotechnology, Santa Cruz, CA, USA; 1:200). After primary antibody incubation, the cells were extensively washed with PBS and incubated with secondary antibodies for 2 h at room temperature. Secondary antibodies were Alexa Fluor 546 (goat anti-rabbit IgG, goat anti-mouse IgG; Molecular Probes, Paisley, UK; 1:1000) or Alexa Fluor 488 (goat anti-rabbit IgG, goat anti-guinea pig IgG, goat anti-mouse IgG; Molecular Probes, Paisley, UK; 1:300). Nuclei were counterstained with DAPI (Sigma Chemical Co., St. Louis, MO, USA). The cells were observed with the aid of a TE2000 Nikon microscope.

#### Presynaptic Activity Analysis

The presynaptic activity assay was performed as previously described (Diniz et al., 2012). Neurons were washed with extracellular solution (150 mM NaCl, 4 mM KCl, 2 mM MgCl_2_, 2 mM CaCl_2_, 10 mM glucose, 10 mM HEPES, pH 7.4) and incubated with a despolarazing solution, a high potassium solution, (97 mM NaCl, 57 mM KCl, 2 mM MgCl_2_, 2 mM CaCl_2_, 10 mM glucose, 10 mM HEPES, pH 7.4) containing FM1-43 (5 μM; Molecular Probes, Paisley, UK) at room temperature for 5 min. Then, neurons were washed with extracellular solution and fixed for observation in a TE2000 Nikon microscope.

#### Synaptical Puncta Analysis

Synapse analysis was performed as previously described (Diniz et al., 2012). Briefly, neurons were randomly identified and selected if nuclei staining (DAPI staining) were, at least, two diameters away from the neighboring neuronal nucleus. Neuronal cultures were analyzed for immunostaining for the pre- and post-synaptic markers, synaptophysin and PSD-95, respectively. The green and red channels were merged and quantified using the Puncta Analyzer plug-in in NIH Image-J as previously described (Diniz et al., 2012). A number of 10–15 images was analyzed and experiments were done in duplicate. Each result represents the mean of at least three independent neuronal cultures.

#### In-Cell Western

Secondary astrocytes were grown in 96 wells-plate for 2 days in DMEM/F12 supplemented with 10% FBS. After reaching confluence, astrocytes were washed and maintained in serum-free medium for 24 h and, then, treated with 10 μM hesperidin or DMSO 0.1% for 2 h. After that, cells were fixed with 4% PFA for 20 min, washed three times with PBS-containing 0.1% triton X-100 and incubated with the Odyssey blocking buffer (LI-COR, Lincoln, NE, USA) for 1.5 h at room temperature. Primary antibodies were: anti-mouse TGF-β1 (Abcam, Cambridge, MA, USA; 1:100) and anti-rabbit cyclophilin B (Sigma Chemical Co., St. Louis, MO, USA; 1:1000) overnight at 4°C. Plates were washed with PBS containing 0.1% tween-20 for three times, followed by incubation with IRDye 680CW goat anti-rabbit and IRDye 800CW goat anti-mouse antibodies (LI-COR, Lincoln, NE, USA; 1:800) for 1 h at room temperature. Plates were scanned with the Odyssey Infrared Imaging System and analyzed using the program Un-Scan-It gel version 6.1 (Silk Scientific, Inc., Orem, UT, USA).

#### Statistical Analysis

Statistical analysis was done by Student’s *t*-test and one-way analysis of variance (ANOVA) followed by Newman-Keuls Multiple Comparison Test, using the Graphpad software version 5.0 (GraphPad Software, La Jolla, CA, USA). A confidence interval of 95% was used, and a *P*-value <0.05 was considered statistically significant. Data are reported as means ± SEM. For each result, exact number of independent experiments and animal samples are described in the legend and in the “Materials and Methods” Section.

## Results

### Hesperidin Promotes Hippocampal Synaptogenesis and Improves Memory in Healthy Mice

Beneficial functions of flavonoids on cognition in animal models for neural disorders have been subjected of several studies (Williams and Spencer, 2012). However, little is investigated about their possible beneficial effects in non-pathological conditions. In order to test whether hesperidin affects memory performance in healthy adult mice, we used a modified protocol from the NOR, by using a longer retention interval between the training and test phase.

**Figure 1:**
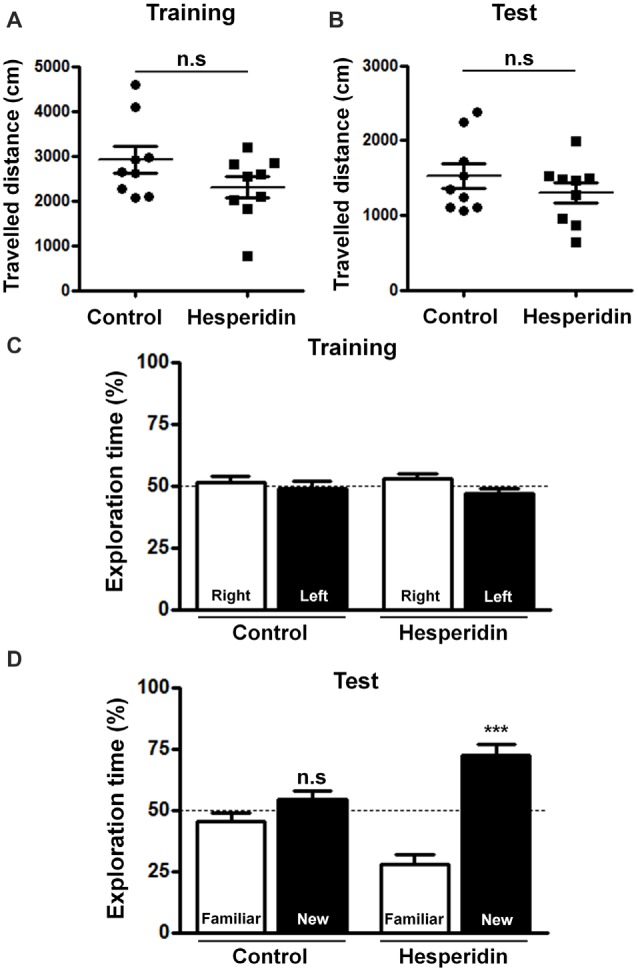
Hesperidin improves memory performance in adult mice. Animals were treated i.p with vehicle (control group) or 10 mg/kg of hesperidin before the training session and, then again, before the object recognition test. Traveled distance during the training **(A)** and the test session **(B)** and recognition memory of objects during training **(C)** and test session **(D)** were measured. Hesperidin significantly improved recognition memory performance of mice. ***p < 0.001, *n* = 9 per experimental group. Student’s test compares the mean exploration time for each object with a fixed value of 50%.

We have not observed any differences in the traveled distance (Figures 1A,B), total exploration time (Supplementary Figures S1A,B) and mean locomotor velocity (Supplementary Figures S1C,D) between the hesperidin and control groups during training and test sessions, indicating that hesperidin has no effect on locomotor/exploratory activities. Additionally, during training, mice from both groups had no preference for the left or right objects (Figure 1C). However, during the test, mice treated with hesperidin spent more time exploring the new object than the familiar one, compared with vehicle treatment (Figure 1D). Results suggest that hesperidin increases the long-term memory performance of healthy adult mice.

**Figure 2:**
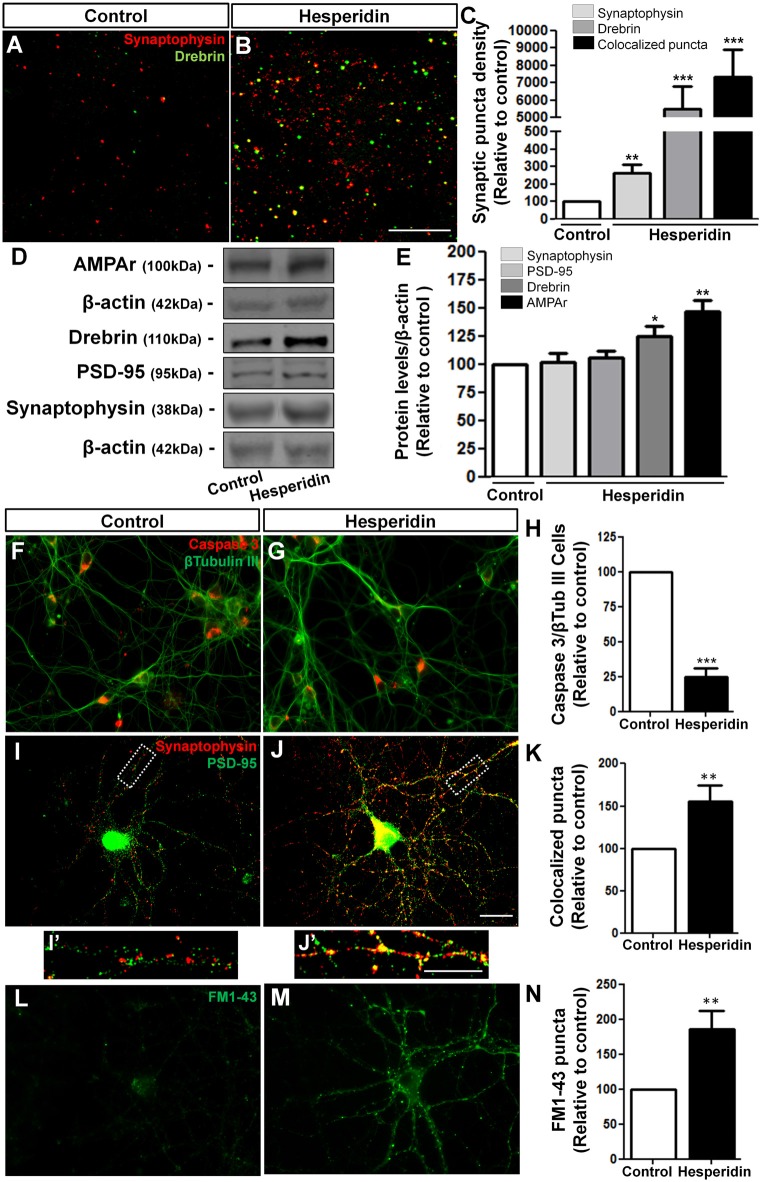
Hesperidin increases synapse formation *in vivo* and *in vitro*. Adult mice treated i.p with vehicle (control group) or 10 mg/kg of hesperidin were submitted to immunohistochemistry analysis for the pre- and post-synaptic markers, synaptophysin and drebrin, respectively **(A–C)** in the hippocampal CA1 area, and western blotting analysis of the levels of the synaptic proteins, synaptophysin, postsynaptic density protein 95 (PSD-95), drebrin and α-amino-3-hydroxy-5- methyl-4-isoxazolepropionic acid receptors (AMPARs) **(D,E)**. Increased synapse formation **(A–C)** and levels of synaptic proteins **(D,E)** were observed in the hippocampus of mice treated with hesperidin. Cortical neurons treated with hesperidin were analyzed by immunocytochemistry for Cleaved Caspase 3 and β-Tubulin-III **(F–H)**, synaptophysin and PSD-95 **(I–K)** and were submitted to the FM1-43 assay **(L–N)**. Neurons treated with the flavonoid showed reduced cell death **(F–H)**, and increased synaptogenesis **(I–K**,**I′**,**J′)**, as well as increased pre-synaptic activity **(L–N)**. Scale bars, 20 μm, 20 μm and 10 μm, respectively. *p < 0.050, **p < 0.010 and ***p < 0.001, *n* = 3–5 animals per experimental group for the *in vivo* experiments and *n* = 3–6 independent neuronal cultures for the *in vitro* experiments. Student’s *t* test.

In order to better understand the underlying mechanisms of hesperidin actions on memory, we analyzed the distribution and levels of synaptic proteins in the hippocampus. We observed that hesperidin remarkably increased synaptophysin and drebrin immunoreactivity (Figures 2A,B), as well as the number of synapses, represented by the colocalization of those two markers, in the hippocampal CA1 area (Figure 2C). These effects were accompanied by a 25% and 46% increase in the total protein levels of drebrin and AMPA receptor in the hippocampus, respectively (Figures 2D,E).

To test the direct action of hesperidin on neurons and synaptogenesis, we first analyzed the effects of hesperidin in the survival of cultured cerebral cortical neurons. To do that, neurons cultured for 3 DIV were then treated for 72 h with hesperidin. Under normal culture condition, the rate of neuronal death is low, approximately 5%. We observed that neurons treated with hesperidin showed increased survival, represented by a reduction in the number of cleaved Caspase 3/β-tubulin III positive cells compared to control (Figures 2F–H). To analyze synapses, 12 DIV neurons were treated for 24 h with the flavonoid. We observed that hesperidin increased the number of double immunostaining puncta for the synaptic markers, synaptophysin and PSD-95, by 55%, suggesting that hesperidin also promotes synapse formation *in vitro* (Figures 2I–K). The synaptogenic effect of hesperidin was not related to an increase in the levels of synaptophysin and PSD-95 proteins *in vitro* (Supplementary Figure S2).

We next investigated whether hesperidin affects the pre-synaptic activity of cultured neurons. To do that, cerebral cortical neurons were treated with hesperidin throughout the 12 DIV and synaptic activity analyzed by the FM1-43 assay. We observed an 85% increase in the number of FM1-43 puncta on neurons treated with hesperidin (Figures 2L–N). Together, these results corroborate the already described neuroprotective function of hesperidin, as well as indicate its novel effect on the synapse formation and function *in vivo* and *in vitro*.

### Hesperidin Increases the Synaptogenic Potential of Astrocytes through the Modulation of Astrocytic TGF-β1 Signaling

**Figure 3:**
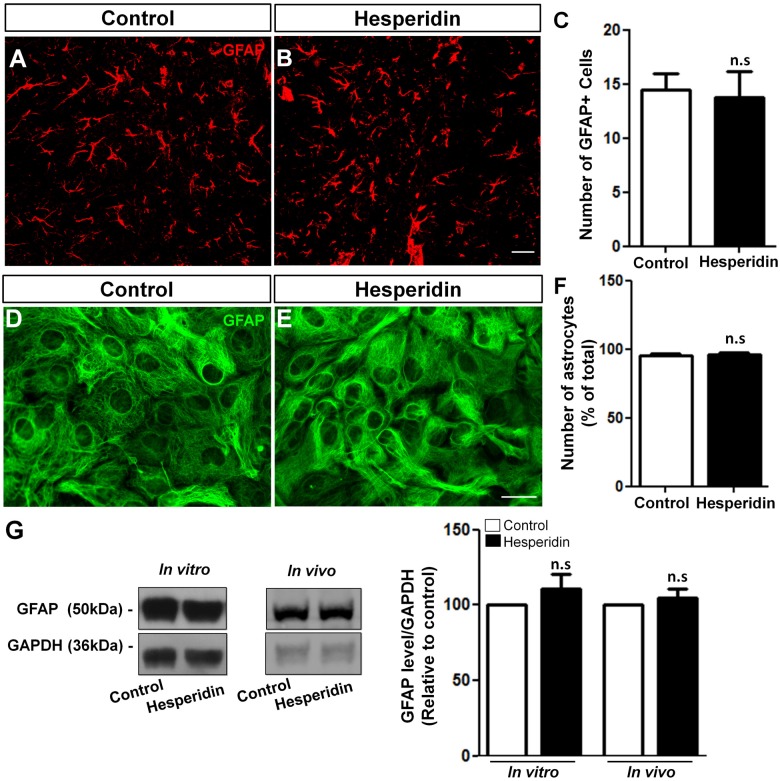
Hesperidin does not affect astrocytes number and glial fibrillary acidic protein (GFAP) levels. Adult mice treated i.p with vehicle or 10 mg/kg of hesperidin were submitted to immunohistochemistry analysis for GFAP **(A–C)** in the hippocampal CA1 area, and western blotting analysis of the level of GFAP **(G)**. Cultured cortical astrocytes were treated with hesperidin and analyzed by immunonocytochemistry **(D–F)** and western blotting for GFAP **(G)**. The number of astrocytes and the levels of GFAP did not change between control and hesperidin groups *in vivo* and *in vitro*. Scale bar, 20 μm, respectively. *n* = 4–6 animals per experimental group and *n* = 3 independent astrocyte cultures. Student’s *t* test.

Although several studies have showed that glial cells are responsive to flavonoids in many experimental disease models (Bahia et al., 2008; Lan et al., 2016), there is a lack of evidences regarding the effects of flavonoids on astrocyte morphology and physiology in the healthy brain. To elucidate this question, we firstly analyzed the effect of hesperidin treatment on astrocytes from the hippocampal CA1 stratum radiatum of adult mice. The quantification of GFAP positive cells revealed that the treatment had no effect on the number of astrocytes (Figures 3A–C), as well as on the level of GFAP in the hippocampus compared to vehicle (Figure 3G).

In addition, we also analyzed the effect of hesperidin in cultured cerebral cortical astrocytes. To do that, astrocytes cultures were treated with 10 μM of hesperidin for 2 h, previously to morphological analysis. Under control and hesperidin treatment conditions astrocytes presented similar flat-protoplasmatic morphology, characteristic of cultured astrocytes (Figures 3D,E). Similarly, number of GFAP positive cells (Figure 3F) and the levels of GFAP protein (Figure 3G) were not affected by the flavonoid. Together, these data suggest that hesperidin, at least in a non-pathological model, does not affect astrocytic morphology and reactivity *in vitro* and *in vivo*.

We previously showed that flavonoids, including hesperidin, enhanced the neuroprotective capacity of astrocytes through an increase in secretion of astrocyte-soluble factors (Nones et al., 2012b). However, there is no evidence regarding whether the synaptogenic ability of astrocytes is affected by flavonoids and the identity of astrocyte-derived soluble factors in this context.

**Figure 4:**
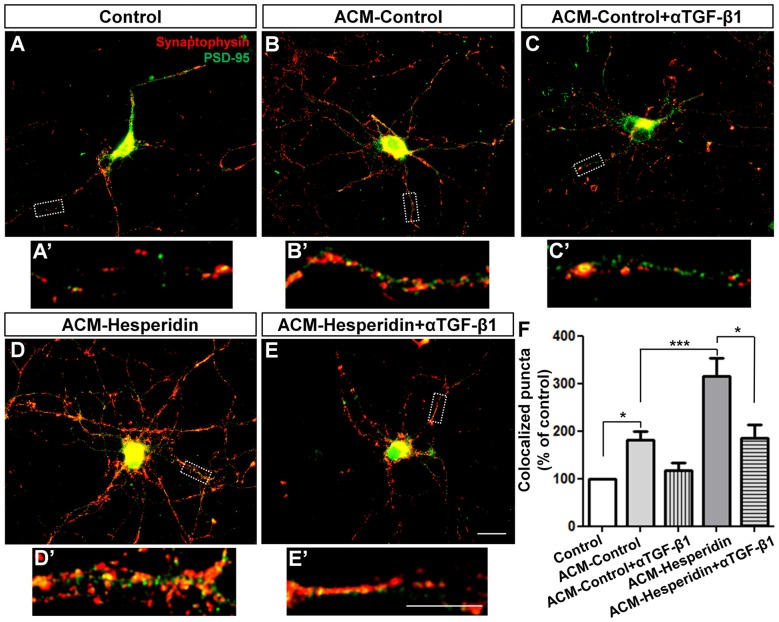
The astrocytic transforming growth factor beta-1 (TGF-β1) mediates the synaptogenic action of astrocytes treated with hesperidin. Twelve days in vitro (12 DIV) cortical neurons were maintained for 3 h in the presence of Dulbecco’s minimum essential medium (DMEM)/F12 (Control; **A**,**A′**), astrocyte conditioned medium (ACM-Control; **B**,**B′**) or astrocyte conditioned medium from astrocytes treated with hesperidin (ACM-Hesperidin; **D**,**D′**), or simultaneously with a neutralizing antibody against TGF-β1 (ACM-Control + αTGF-β1; **C**,**C′**, and ACM-Hesperidin + αTGF-β1; **E**,**E′**)synapse formation was evaluated by immunocytochemistry for the synaptic markers, synaptophysin and PSD-95. ACM-Control increased the number of synapses by two times relative to control and ACM-Hesperidin enhanced synapse formation by two times in relation to ACM-Control; whereas depletion of TGF-β1 partially blocked this effect. Scale bars 20 μm **(E)** and 10 μm **(E′)**. *p < 0.050 and ***p < 0.001; comparisons among multiple groups were analyzed using a one-way analysis of variance (ANOVA) followed by Newman-Keuls *post hoc* tests. *n* = 3–6 independent astrocyte cultures.

TGF-β1 is a pleiotropic factor expressed by astrocytes from different brain regions (Buosi et al., 2017), widely secreted by cortical astrocytes and involved in synapse formation and function (Diniz et al., 2012, 2014b; Araujo et al., 2016). To investigate whether hesperidin affects the synaptogenic ability of astrocytes, we treated 12 DIV neuronal cultures with the ACM-Control or ACM-Hesperidin for 3 h, and then analyzed the number of synapses. ACM-Control increased the number of synapses by 80% (Figures 4A,B,F). Surprisingly, ACM-Hesperidin was more effective in promoting synapse formation than ACM-Control, with a two-fold increase in relation to ACM-Control (Figures 4B,D,F).

To verify the involvement of TGF-β1 in the synaptogenic ability of astrocytes, we pre-incubated the ACMs with neutralizing antibody against TGF-β1 (αTGF-β1) before the treatment of neuronal cultures with the ACMs. Although we have observed a decreased synaptogenic effect of ACM-Control incubated with αTGF-β1, this effect was not statistically significant (Figures 4C,F). However, effect of ACM-Hesperidin was strongly reduced by addition of αTGF-β1 (Figures 4D–F). These results suggest that hesperidin improves the synaptogenic potential of astrocytes and points to TGF-β1 as an important synaptogenic factor secreted by astrocytes in response to hesperidin.

**Figure 5:**
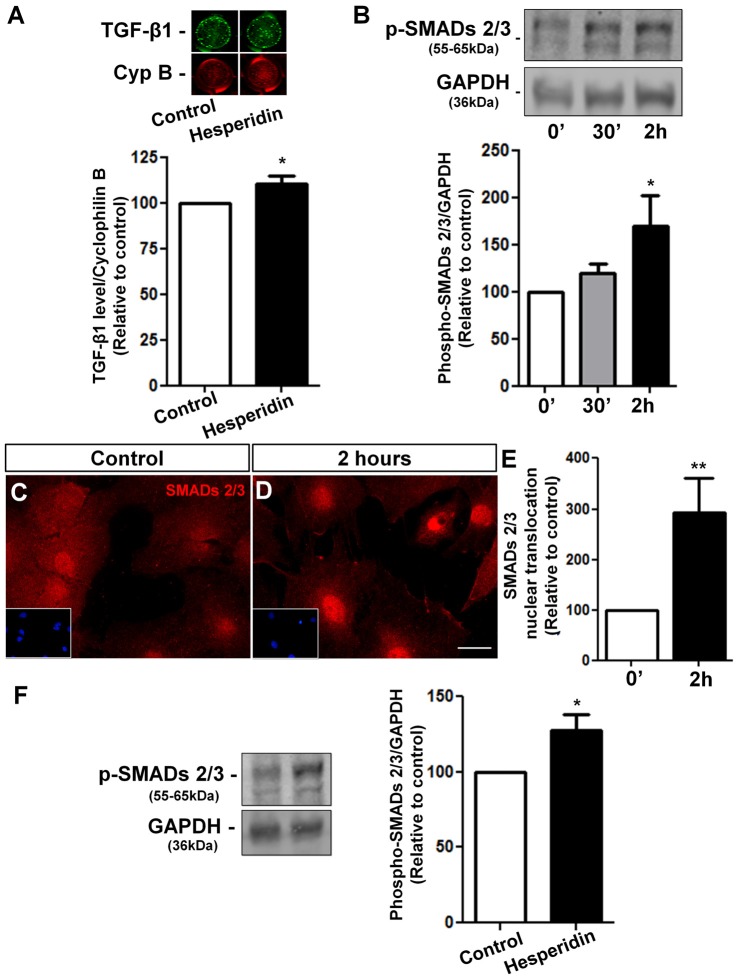
Hesperidin increases TGF-β1 levels and activates its signaling pathway in cultured astrocytes and in the hippocampus. Cortical astrocytes were treated with hesperidin for 2 h and submitted to In-Cell western analysis for TGF-β1 **(A)**. Activation of TGF-β1 signaling pathway was analyzed by Western blotting assays for phospho-SMADs 2/3 **(B)** and by evaluation of nuclear translocation of SMADs 2/3 **(C–E)**. Levels of phospho-SMADs 2/3 were also analyzed in the hippocampus of adult mice treated with vehicle or hesperidin **(F)**. Hesperidin slightly increases the levels of astrocytic TGF-β1 and strongly induced phosphorylation and activation of the SMADs 2/3 signaling pathway *in vitro* and *in vivo*. *p < 0.050 and **p < 0.010. Student’s *t* test was used in **(A,E,F)**. Comparisons among multiple groups were analyzed using a one-way ANOVA followed by Newman-Keuls *post hoc* test in **(B)**. *n* = 4–5 independent astrocyte cultures for the *in vitro* experiments and *n* = 4–6 animals per experimental group for the *in vivo* experiments.

In order to address the underlying mechanisms of hesperidin action on the synaptogenic potential of astrocytes, we treated confluent astrocyte cultures with hesperidin for 2 h and analyzed the levels of TGF-β1in these cells. We observed a slightly, but significant, increase in the level of TGF-β1 in astrocytes in response to hesperidin (Figure 5A).

To investigate whether hesperidin activates the TGF-β/SMADs signaling pathway, we performed kinetics assays in astrocytes treated with hesperidin for 0, 30 min and 2 h. We found a 70% increase in the phospho-SMADs 2/3 levels in astrocytes treated for 2 h with the flavonoid (Figure 5B). These findings were supported by the increased nuclear translocation of SMADs 2/3 in hesperidin treated-astrocytes, a hallmark of TGF-β/SMADs pathway activation (Figures 5C–E). Further, we also observed a 30% increase of phospho-SMADs 2/3 levels in the hippocampus of mice injected with hesperidin (Figure 5F).

Together, these results indicate that the TGF-β1 signaling is modulated by hesperidin *in vitro* and *in vivo* and TGF-β1 is one of the indentified molecules that mediates the synaptogenic action of hesperidin-treated astrocytes.

## Discussion

In the current study, we described the beneficial effects of the short-term treatment with hesperidin on memory of healthy adult mice and its underlying cellular mechanisms. We showed that hesperidin promotes synaptogenesis and increases the levels of synaptic proteins in the hippocampus. We further elucidated its cellular targets and mechanisms using *in vitro* approaches and we found that hesperidin exerts two distinct effects on synapses: a direct effect, by increasing synapse formation and pre-synaptic activity; and an indirect effect, by enhancing the synaptogenic potential of astrocytes through TGF-β1 secretion and activation of TGF-β1-SMADs 2/3 signaling.

Evidences suggest that long-term ingestion of flavonoid-rich food potentially prevents age-related cognitive decline in humans and in several murine models (Kean et al., 2015; Matias et al., 2016). Most of the data are related to the potential of flavonoids to prevent cognitive deficits or rescue learning and memory impairments in pathological contexts, including animal models for Alzheimer’s (Gu et al., 2016), Parkinson’s (Antunes et al., 2014) and Huntington’s diseases (Sandhir and Mehrotra, 2013) and in Multiple Sclerosis (Makar et al., 2016). Nevertheless, it has been showed that these compounds may also improve the cognitive ability of healthy young and old animals (van Praag et al., 2007; Williams et al., 2008; Rendeiro et al., 2013). Despite of this, cellular and molecular mechanisms of flavonoid’s actions in these cases remain poorly understood, especially in relation to their effects in the healthy brain.

The most well described mechanism by which flavonoids can ameliorate or rescue cognitive ability is through the modulation of synaptic plasticity and function. *In vivo* evidences have showed that the chronic administration of 7,8-dihydroxyflavone improved spatial memory and mitigated dendritic spine and AMPARs loss in the hippocampus of a mouse model for Alzheimer’s disease (Gao et al., 2016). Similar results have been reported in different animal models for neural diseases and in cognitively impaired aged rats treated chronically with flavonoids (Zeng et al., 2012; Tian et al., 2015; Gu et al., 2016). However, there are fewer evidences concerning the effects of flavonoids on synapses in non-pathological models.

Here, we showed that the memory performance improvement of healthy mice treated acutely with hesperidin were accompanied by a higher number of structural synapses in the hippocampal CA1 area and protein levels of drebrin and AMPARs in their hippocampus. In agreement with our data, studies have demonstrated that the dietary supplementation of flavonoids, particularly anthocyanins and flavanols, enhances spatial memory performance of healthy mice, events related to increased levels of hippocampal BNDF (Williams et al., 2008; Rendeiro et al., 2013). Interestingly, increased levels of NMDA-NR2B receptor have also been showed in the hippocampus of healthy young rats treated with a mix of anthocyanins and flavanols (Rendeiro et al., 2014). Therefore, our results reinforce the involvement of glutamatergic receptors and downstream signaling pathways, as well as the proper reorganization of pre- and post-synaptic proteins that could strength hippocampal connectivity and facilitate LTP in the effects of flavonoids on synapse formation.

Although *in vivo* evidences have shown many beneficial effects of hesperidin on the CNS, its cellular targets and pathways still need to be elucidated. Here, we showed that hesperidin promoted survival and synapse formation and activity between cultured cortical neurons. Our previous data indicated that hesperidin increases neurogenesis by inducing differentiation of neural progenitors and supports neuronal survival *in vitro* (Nones et al., 2011). Whereas the neuroprotective property of hesperidin has been extensively investigated (Menze et al., 2012; Hong and An, 2015), its actions on synapses are less known. A screening of 65 flavonoids showed that hesperidin, among eight flavanones evaluated, was the only one able to increase the expression of synaptotagmin, but not PSD-95, in cortical neurons *in vitro* (Xu et al., 2013b). Here, we analyzed the total levels of synaptic proteins in neurons treated with hesperidin, and, although we have not observed differences in the levels of synaptophysin and PSD-95, we observed a higher number of synapses and pre-synaptic activity between cells, suggesting that the flavonoid modulates synapse formation and possibly synaptic function *in vitro*.

Given the remarkable role of astrocytes on synapse formation, maintenance and function, a better characterization of flavonoids actions on these cells would contribute to our knowledge about how synapses form and function. In the current study, we showed that hesperidin does not change the number of astrocytes and levels of GFAP in the hippocampus of adult mice neither in culture. These data are apparently against those obtained from murine models for age-related diseases. Under pathological contexts, flavonoids may attenuate the inflammatory profile of astrocytes, as observed by reduced expression of GFAP gene and down-regulation of pro-inflammatory pathways (Currais et al., 2014; Rehman et al., 2017). Previous data from our group, however, also showed that another flavonoid, casticin, does not affect morphology and proliferation rate of cortical astrocytes (de Sampaio e Spohr et al., 2010). Therefore, these data suggest that the morphological profile of astrocytes is not modified by flavonoids under non-pathological conditions. In contrast, in the injured brain, flavonoids may rescue physiological phenotype of these cells (Sharma et al., 2007) including up-regulation of their antioxidant capacity (Bahia et al., 2008; Park et al., 2011), events that were related to prevention of neuronal cell death *in vitro* (Vafeiadou et al., 2009; Nones et al., 2012b) and that contribute to the rescue of cognitive function in disease models (Currais et al., 2014; Rehman et al., 2017).

One of the main mechanisms by which astrocytes modulate neuronal connectivity and function is through the secretion of trophic factors (Diniz et al., 2014a). We and other workers have shown that flavonoids may modulate the secretion of neurotrophic factors by astrocytes (de Sampaio e Spohr et al., 2010; Nones et al., 2012b; Xu et al., 2013a), although the identity of these factors is not fully known. A screening of thirty-three flavonoids, including hesperidin, showed that most of them are able to increase the astrocytic levels of NGF, GDNF and BDNF secretion (Xu et al., 2013a). In agreement, *in vivo* studies showed that the long-term treatment of adult mice with hesperidin results in higher levels of hippocampal BDNF, an event related to the antidepressant-like effect of hesperidin (Donato et al., 2014; Antunes et al., 2016). Here, we demonstrated that hesperidin increased the synaptogenic potential of cortical astrocytes *in vitro* by inducing TGF-β1 synthesis and activation of its signaling pathway and in the hippocampus of adult mice.

TGF-β1 is a pleiotropic cytokine involved in several steps of brain development and function, including astrocyte generation and synapse formation (Diniz et al., 2014a; Stipursky et al., 2015). TGF-β1 signaling is expressed by astrocyte progenitors in the cerebral cortex during brain development (Stipursky and Gomes, 2007; Stipursky et al., 2012) and by mature astrocytes in different brain regions during synaptogenic period (Araujo et al., 2016; Buosi et al., 2017). Recently, we demonstrated that astrocytes control the balance between excitatory and inhibitory synapses in the cerebral cortex by activating distinct downstream TGF-β1 pathways (Diniz et al., 2012, 2014b; Araujo et al., 2016). Here, we demonstrated that TGF-β1 is essential for the increased synaptogenic potential of hesperidin-treated astrocytes. This is supported by the observation that TGF-β1 neutralizing antibody assays impaired the synaptogenic property of the hesperidin-treated astrocytes. Further, we also showed that hesperidin activates signaling pathway of the astrocytic TGF-β1. Together, we firstly provided evidence that flavonoids may improve the synaptogenic ability of astrocytes through an up-regulation of secreted-soluble factors known to be involved in synapse formation and function.

Since the effect of hesperidin on the astrocytic SMADs 2/3 activation was time-dependent, we suggest that the flavonoid does not directly binds or actives the TGF-β receptor, but rather, it probably triggers an indirect effect through the modulation of non-canonical pathways of TGF-β. The crosstalk between TGF-β with other signaling pathways is well described, such as with the mitogen-activated protein kinases (MAPKs) pathways. In these cases, activation of TGF-β receptors leads to downstream activation of MAPKs; inversely, these kinases can also regulate SMADs phosphorylation (Funaba et al., 2002; Derynck and Zhang, 2003). Indeed, previous data from our group showed that hesperidin is able to activate neuronal MAPK and PI3K pathways, which are involved in the hesperidin neuroprotective action (Nones et al., 2012a). In agreement, other flavanones, such as hesperitin and 5-nitro-hesperitin, have been described to prevent neuronal death through the activation of pro-survival Akt and ERK1/2 in cortical neurons (Vauzour et al., 2007). Together with our results, these evidences raise the possibility that hesperidin positively modulates the astrocytic TGF-β1/SMADs 2/3 signaling through involvement of non-canonical pathways. The full elucidation of this mechanism will deserve further investigation.

While most of the evidences concerning flavonoids’ actions on the CNS refer to the long-term treatment or dietary intake of flavonoids, here, we shed light on the beneficial effects of these compounds in a short-term intervention. In this perspective, flavonoids present the potential to be useful as pharmacological drugs capable to directly modulate CNS function and repair. Collectively, we described hesperidin as a new molecule able to improve the recognition memory performance in healthy adult mice, an event accompanied by an increase in hippocampal synapse formation. *In vitro* assays indicated that hesperidin exerts such effects on synapse through 2 distinct mechanisms: direct on neurons and indirect, by enhancing astrocytic synaptogenic potential and TGF-β1 signaling in these cells. These results strengthen the potential of flavonoids as new therapeutic approaches for the prevention and/or treatment of neurological disorders, which are accompanied by synaptic dysfunction and loss of memory and point to the potential of astrocytes as targets for these natural compounds.

## Author Contributions

IM, LPD, AB, GN, JS and FCAG: designed experiments. IM, LPD and AB: performed experiments. IM, LPD, AB, GN, JS and FCAG: analyzed data. IM, GN, JS and FCAG: wrote the article.

## Conflict of Interest Statement

The authors declare that the research was conducted in the absence of any commercial or financial relationships that could be construed as a potential conflict of interest.

## Abbreviations

ACM: astrocyte-conditioned medium
AMPAR: α-amino-3-hydroxy-5-methyl-4-isoxazolepropionic acid receptor
CNS: central nervous system
DAPI: 4′,6-diamidino-2-phenylindole dihydrochloride
DIV: days *in vitro*
DMEM/F12: Dulbecco’s modified Eagle’s medium
DMSO: dimethyl sulfoxide
FBS: fetal bovine serum
GAPDH: glyceraldehyde-3-phosphate dehydrogenase
GFAP: glial fibrillary acidic protein
NOR: novel object recognition test
PBS: phosphate-buffered saline
PFA: paraformaldehyde
PSD-95: postsynaptic density protein 95
TGF-β1: transforming growth factor beta-1.
