# Supplementary figures and images for: Flavonoid Hesperidin Induces Synapse Formation and Improves Memory Performance through the Astrocytic TGF-β1
Source: Front Aging Neurosci. 2017 Jun 13;9:184. doi: 10.3389/fnagi.2017.00184 (PMC5468382; doi:10.3389/fnagi.2017.00184)

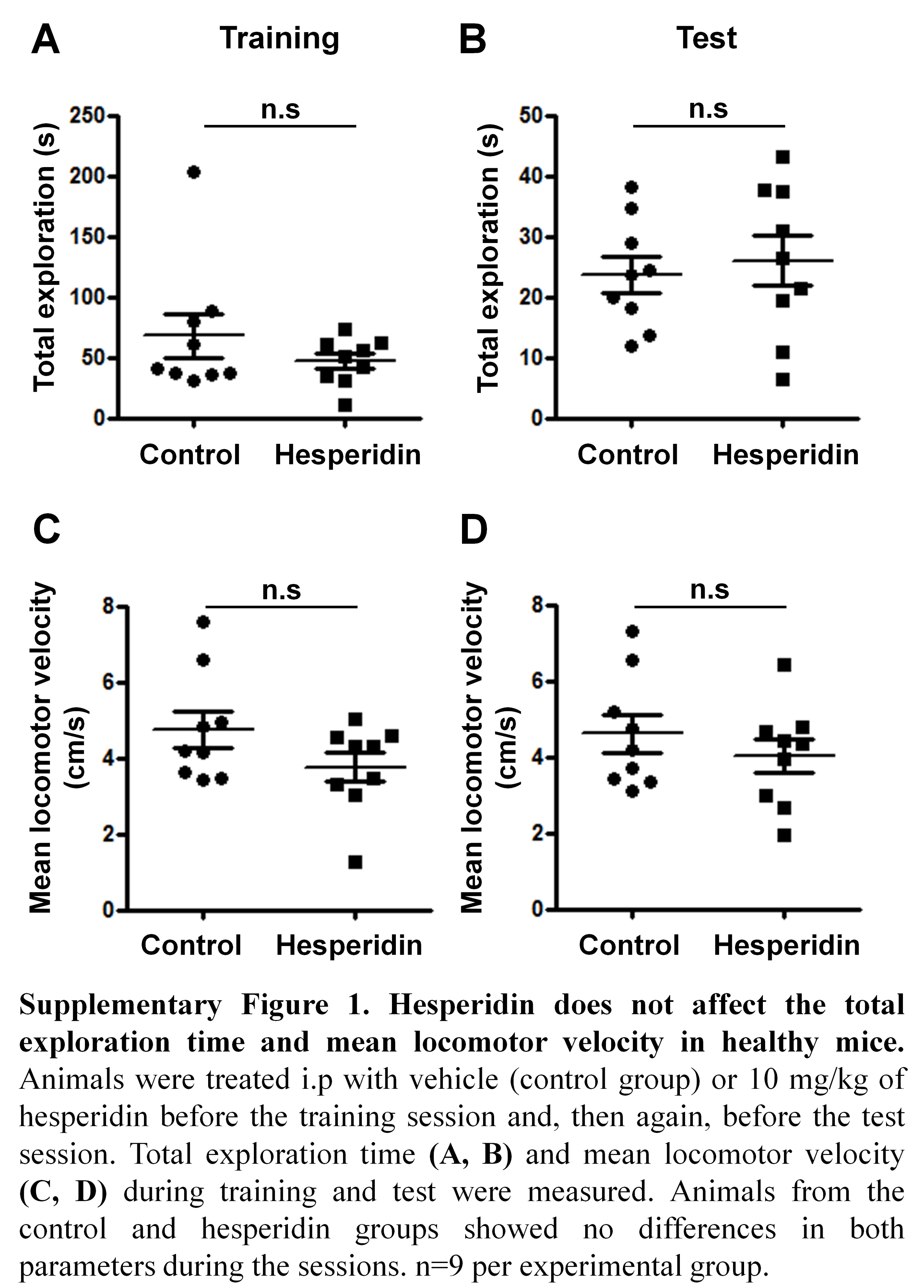

Supplement: Supplementary file 1 [file Image_1.tif]

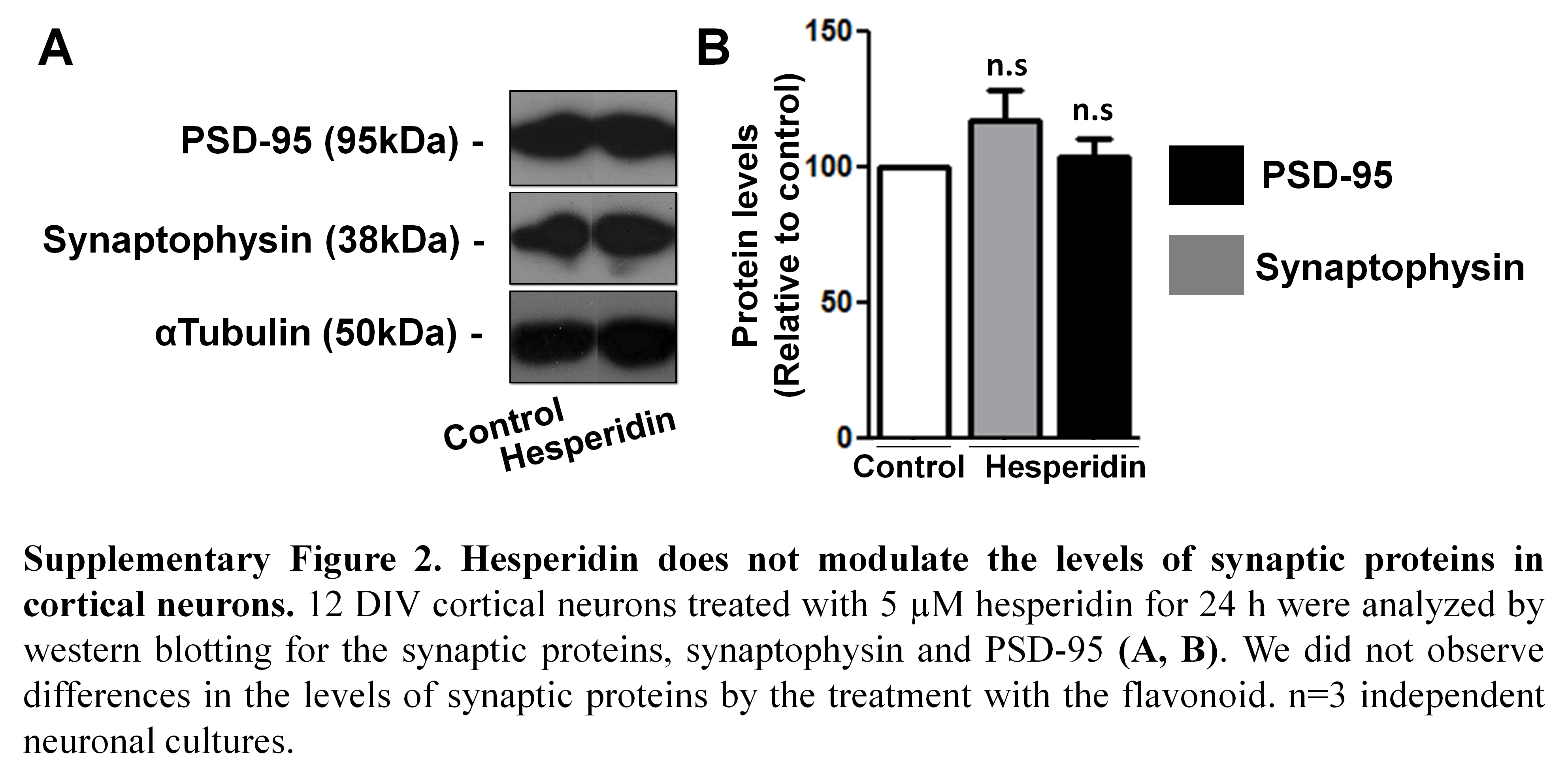

Supplement: Supplementary file 2 [file Image_2.tif]
